# Christmas Tree-Shaped Microneedles as FOLFIRINOX Spatiotemporal Delivery System for Pancreatic Cancer Treatment

**DOI:** 10.34133/2022/9809417

**Published:** 2022-10-16

**Authors:** Danqing Huang, Xiao Fu, Xiaoxuan Zhang, Yuanjin Zhao

**Affiliations:** ^1^Institute of Translational Medicine, The Affiliated Drum Tower Hospital of Nanjing University Medical School, Nanjing 210002, China; ^2^Oujiang Laboratory (Zhejiang Lab for Regenerative Medicine, Vision and Brain Health), Wenzhou Institute, University of Chinese Academy of Sciences, Wenzhou, Zhejiang 325000, China; ^3^State Key Laboratory of Bioelectronics, School of Biological Science and Medical Engineering, Southeast University, Nanjing 210096, China

## Abstract

As an effective combination chemotherapy, FOLFIRINOX regimen (fluorouracil, leucovorin, irinotecan, and oxaliplatin) has shown definite antitumor efficacy for treating pancreatic cancer (PC) nowadays. However, the traditional systematic administration of these chemotherapeutics limits the drug targeting and causes unwanted effects. Herein, we present a novel Christmas tree-shaped adhesive microneedle (MN) patch coloading fluorouracil, leucovorin, irinotecan, and oxaliplatin simultaneously to realize spatiotemporal FOLFIRINOX therapy in situ. Such MN patch was fabricated by using a layer-by-layer mold replication method, in which oxaliplatin and leucovorin are encapsulated in the top MNs, while irinotecan and fluorouracil are encapsulated in the bottom MNs. The multilayer structure imparts the MNs with enhanced adhesive ability and spatiotemporal drug release property, contributing to the antitumor effect on PC organoid models. Therefore, our Christmas tree-shaped MN patch represents an innovative approach for spatiotemporal multiple-drug delivering and realizes the combination chemotherapy for PC in a single platform.

## 1. Introduction

Pancreatic cancer (PC) is a refractory disease with an increasing morbidity [1, 2]. The 5-year survival of PC patients is less than 10% because over 80% of them are diagnosed at late stages, which benefit little from surgery [3, 4]. Relatively, chemotherapy is applied on most PC patients and has been approved to be effective to treat PC. In recent decades, it has become a consensus that the antitumor efficacy of combination chemotherapy is better than that of a single drug [5–7]. Among the developed regimes, the FOLFIRINOX regimen has been demonstrated as the most effective treatment for PC, which refers to a combination of oxaliplatin (L-OHP), leucovorin (LV), irinotecan (CPT-11), and fluorouracil (5-FU) [8–10]. To employ FOLFIRINOX regimen, the therapeutic procedure includes a sequential injection of L-OHP, LV, and CPT-11, followed by a 46-hour intravenous infusion of 5-FU. Although with some successes, the systematic administration of chemotherapeutics and the augmented desmoplasia in PC severely compromise the therapeutic effect and cause a lot of adverse events [11–13]. In addition, many attempts have been made to enhance the drug targeting in PC, such as binding paclitaxel with albumin and encapsulating CPT-11 with liposome, but the pancreatic tumor inhibitory effect is still far from satisfactory [14, 15]. Thus, a new method that can target the tumor tissue, enhance drug delivery, and realize FOLFIRINOX regimen is still anticipated.

**Figure 1:**
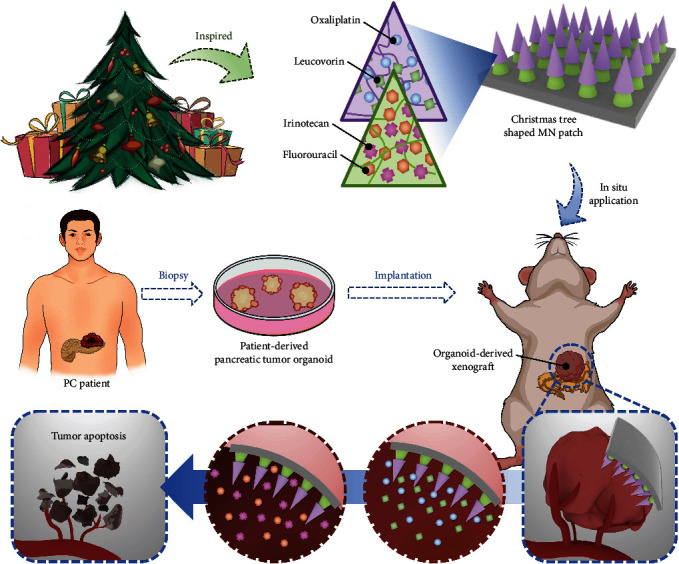
Design and application of the Christmas tree-shaped MN patch. The Christmas tree-inspired MN patch contained two layers of MNs, where different chemotherapeutics can be loaded in different MNs. The biodegradability and anticancer efficacy of the FOLFIRINOX-loaded Christmas tree-shaped MN patch on patient-derived pancreatic cancer organoids.

Herein, we present a novel Christmas tree-shaped microneedle (MN) patch coloading L-OHP, LV, CPT-11, and 5-FU simultaneously to realize spatiotemporal FOLFIRINOX therapy in a single regimen for the PC treatment, as schemed in Figure 1. Attributing to the micron-scale and outstanding drug-loading efficiency, MNs have been designed for transdermal and relatively painless drug administration [16–18]. To date, MNs are no longer limited to treat superficial wounds or diseases, and considerable progresses have been achieved in curing internal diseases, including myocardial ischemia and abdominal tumors [19, 20]. To impart MNs with diverse functions, various biocompatible and biomodifiable materials can be employed, such as gelatin methacryloyl (GelMA), poly (ethylene glycol) diacrylate (PEGDA), and poly (N-isopropylacrylamide) [21–23]. Attractively, by modulating the polymer composites and microarchitectures of the MNs, desired drug release kinetics and mechanical properties can be achieved [24–26]. However, since the four chemotherapeutics applied in FOLFIRINOX therapy are required to be administrated in a spatiotemporal manner, the MN patch which delivers only single drug is not sufficient to achieve the therapeutic efficacy. This together with the moist abdominal environment and irregular surface of the pancreatic tumor indicated that specially designed MNs with desired spatiotemporal drug release kinetics and adequate anchoring strength were required.

Thus, inspired by the hierarchical architecture of Christmas tree, we fabricated a multilayer MN patch with L-OHP and LV encapsulated in the top MNs, while CPT-11 and 5-FU encapsulated in the bottom MNs, realizing the spatiotemporal administrated FOLFIRINOX therapy in a single platform to treat PC. Benefitting from the biocompatibility and maneuverability of GelMA and PEGDA, we testified the drug loading efficiencies, release kinetics, and mechanical properties, thus optimizing ideal concentrations of GelMA and PEGDA to fabricate the two layers of our MNs. It was demonstrated that the drugs encapsulated in the top and bottom MNs could be released sustainably in batches, imitating the administration of FOLFIRINOX in clinical practice. Additionally, compared with the traditional single-layer MN patch, our Christmas tree-shaped MN patch showed a significantly enhanced adhesive property, contributing to the stable adhesiveness to the moist and irregular surface of abdominal tumors. Furthermore, we illustrated the cytotoxicity of the Christmas tree-shaped MNs on PC organoids. By employing an organoid derived xenograft model established on mice, our MNs showed significant advantages over the traditional administration of FOLFIRINOX. Therefore, the proposed multidrug spatiotemporal delivery Christmas tree-shaped MNs suggested an effective stratagem for treating PC.

## 2. Results

**Figure 2:**
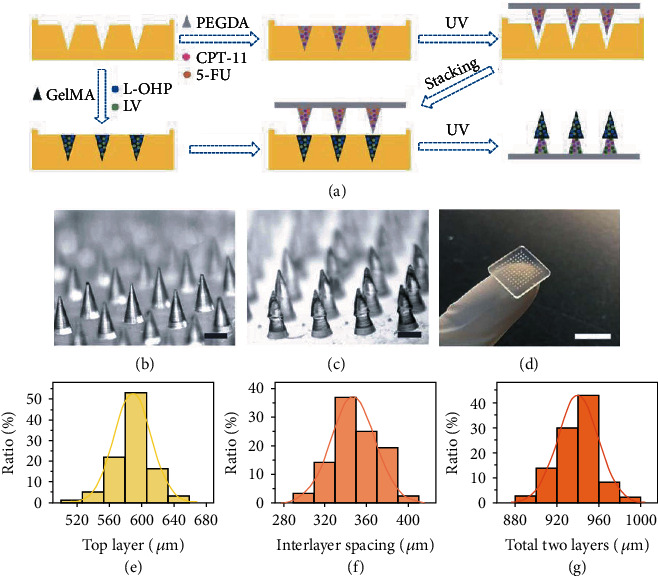
Fabrication process and morphology characterization of the Christmas tree-shaped MN patch. (a) Schematic flow gram of the fabrication process of the FOLFIRINOX-loaded Christmas tree-shaped MN patch. (b, c) Optical microscopic images of the monolayer MN patch (b) and Christmas tree-shaped MN patch (c). (e–g) Statistical quantification of the height of the top layer (e), interlayer spacing (f), and total two layers (g). Scale bars are 500 *μ*m in (b, c) and 1 cm in (d).

In a typical experiment, the FOLFIRINOX-loaded Christmas tree-shaped MN patch was fabricated based on the mold replication process (Figure 2(a)). The pregel PEGDA solution mixed with CPT-11 and 5-FU (for bottom layer) was filled into a negative mold with orderly arranged conical cavities (Figure S1). The excess pregel solution was removed before the solidification via ultraviolet (UV) light. Then, extra empty loaded PEGDA prepolymer solution was added to the negative model containing bottom layer MNs to offer a base patch. After the UV solidification of the bottom patch (Figure 2(b)), pregel GelMA solution mixed with L-OHP and LV (for top layer) was filled into another negative MN model. After the removal of the extra pregel solution, the bottom MN patch was pressed slightly into the negative model, followed by the irradiation of UV light. Thus, the top layer and the bottom patch were contacted tightly, and the resultant Christmas tree-shaped MN patch can be obtained (Figures 2(c) and 2(d)). The Christmas tree-shaped MNs showed uniform morphology, height, and interlayer spaces. Statistical analyses of the height of top layer, interlayer spacing, and total two layers of the Christmas tree-shaped MNs showed the batch stability of the fabrication process (Figures 2(e)–2(g)). The two-layer microstructure of our Christmas tree-shaped MNs simulated the hierarchical microarchitecture of the stings of wasps, intending to enhance the adhesive ability of the MN patch.

**Figure 3:**
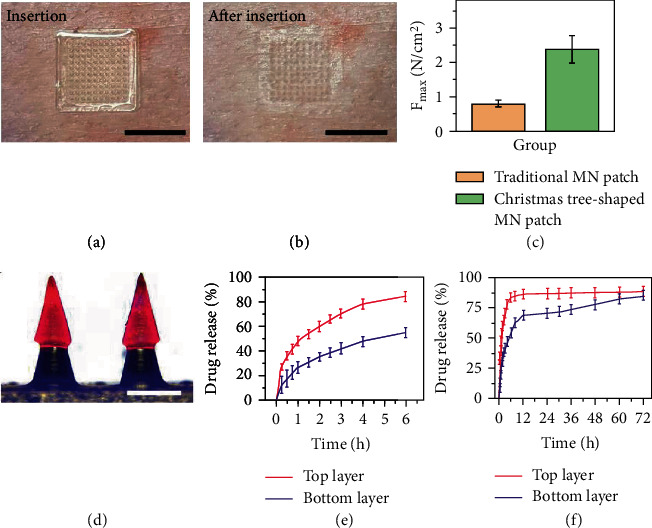
Adhesive property and drug release kinetics of the Christmas tree-shaped MN patch. (a) Application of the Christmas tree-shaped MN patch to swine skin. (b) After pressing the MN patch on the pork for 1 min, micropores can be found on the pork after removal. (c) Comparisons of the maximum detachment forces of the traditional single-layer MN patch and the Christmas tree-shaped MN patch from swine skin. (d) The merge image of the Christmas tree-shaped MN patch. (e, f) Drug release characterization of the FOLFIRINOX-loaded MNs in 6 h (e) and 72 h (f). Scale bars are 1 cm in (a, b) and 500 *μ*m in (d).

Since the hierarchical morphologies of the Christmas tree-shaped MNs have been replicated, it is of great significance to evaluate the tissue adhesion property of the Christmas tree-shaped MN patch. First, we applied a piece of porcine skin to assess the tissue penetration of the MNs. After the successful fabrication of the two-layer MN patch, it was placed on the surface of the porcine skin with the needle tips facing the skin. Then, the MN patch was pressed with finger for at least 1 min before the removal (Figure 3(a)). As illustrated in Figure 3(b), the mechanical property of GelMA hydrogel allowed the penetration through tissues, and periodical arrayed micropores can be observed on the swine skin. To exhibit the advantages of the Christmas tree-shaped MNs in adhesion, we further compared the detachment forces between the traditional one-layer MN patch and the Christmas tree-shaped two-layer MN patch. During the experimental process, a piece of porcine skin with slippery and wet surface was employed, which could better imitate the internal environment of the human body. Statistical results showed that the two-layer MN patch featured an obvious enhanced adhesive property (Figure 3(c)). Attributing to the unique morphology of the Christmas tree-shaped MN tips, these MN tips can tightly pierce into the tissue. Since the enhanced adhesive property was benefited from the physical interlocking between the two-layer MNs and tissue, the slippery and wet surface of the tissue would not affect the adhesion efficacy, which paved the way for the further applications.

The evaluation of the spatiotemporal drug loading and release kinetics was then carried out. In clinical practice, the FOLFIRINOX regime consisted of 4 parts, including a 2-hour intravenous infusion of L-OHP, a 2-hour intravenous infusion of LV, a 90-minute intravenous infusion of CPT-11, and a 46-hour intravenous infusion of 5-FU. Thus, the initial sudden release of L-OHP, LV, and CPT-11 and the sustained release 5-FU are required to achieve in the MN platform. In the typical experiment, water-soluble red and blue molecular fluorescent dyes were loaded in the top and bottom MNs, respectively. As shown in Figure 3(d) and Figure S2, the bottom MNs emitted blue fluorescence while the top MNs emitted red fluorescence, indicating the spatial drug-loading capacity of the MNs in different layers. Thus, it was feasible to realize the FOLFIRINOX therapy through encapsulating L-OHP and LV in the top MNs and encapsulating CPT-11 and 5-FU in the bottom MNs. Furthermore, we measured the temporal drug release capacity of the multi-drug-loaded MNs. According to the detectable absorbance of L-OHP and 5-FU, these two chemotherapeutics were chosen to represent the 72 h release kinetics of drugs loaded in top and bottom MNs, respectively. As illustrated in Figure 3(e), CPT-11 showed a sudden and massive release in the first 3 h. In contrast, 5-FU showed relatively small release in the first 3 h, and a prolonged and sustained release can be measured over the next 72 h (Figure 3(f)). The specific spatiotemporal drug release kinetics could imitate the clinical administration of FOLFIRINOX regime, paving way for the antitumor efficacy in the downstream experiments.

**Figure 4:**
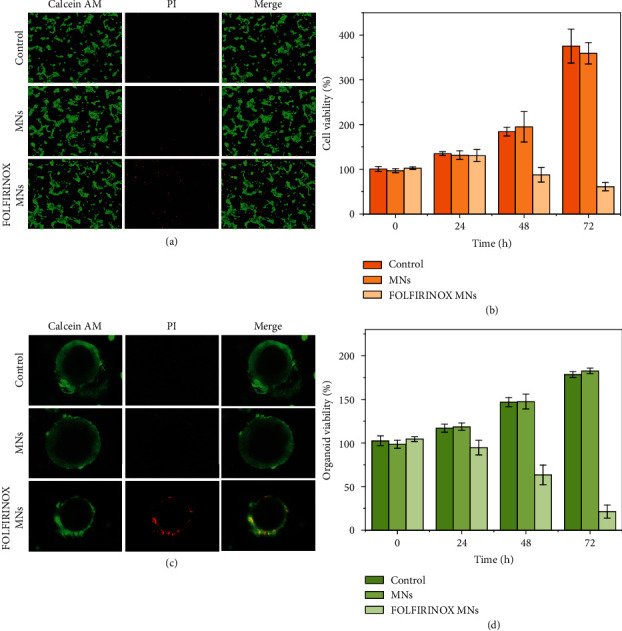
Cytotoxicity of the FOLFIRINOX-loaded Christmas tree-shaped MNs on Capan1 cells and PC organoids. (a) Live and dead staining of the Capan1 cells from different groups. (b) Statistical results of the relative cell viability after different treatment at 0, 24, 48, and 72 h. (c) Live and dead staining of the patient-derived pancreatic tumor organoids. (d) Statistical results of the relative organoid viability after different treatments at 0, 24, 48, and 72 h. Scale bar is 50 *μ*m in (a) and (c).

We evaluated the biocompatibility of the MN patch on 3T3 cells first (Figure S3). The cells showed normal and healthy morphology after 24 h coincubation with MNs, which had no significant discrepancy with the cells in the control group, indicating the excellent biosafety of our Christmas tree-shaped MNs. Then, PC cell line Capan1 was employed to verify the cytotoxicity of the FOLFIRINOX MNs. The Capan1 cells were randomized into three groups, namely, the control group, the MN group, and the FOLFIRINOX MN groups. According to the results of drug release experiments, the FOLFIRINOX MNs were aimed at realizing the clinically practiced FOLFIRINOX regimen, leading to desired cancer cell killing effect. Consistent with our theory, by staining the cells with Calcein AM&PI, the Capan1 cells showed healthy morphology (green fluorescence) in the control and MN group, while significantly increased dead cells (red fluorescence) can be observed in the FOLFOIRINOX MN group after 48 h treatment (Figure 4(a)). Identically, the cell viability measured by the cell counting kit-8 (CCK-8) from different groups after 24, 48, and 72 h treatment was consistent with the live and dead staining results (Figure 4(b)). The inhibited Capan1 cell viability can be detected after 48 and 72 h treatment, implying the prolonged cytotoxicity of FOLFIRINOX MNs.

In recent years, in vitro organoid culturation has shown broad potential in imitating in vivo physiological functions [27, 28]. Specifically, tumor organoids can exhibit biological characteristics and behaviors more similar to those of tumors in vivo [29, 30]. Thus, we herein employed patient-derived PC organoids for the evaluation of the antitumor efficacy of the FOLFIRINOX MNs. Based on the biopsy tumor samples from patients that have been diagnosed with PC, pancreatic tumor organoids can be cultured in Matrigel. After dividing the organoids into the control group, MN group, and FOLFIRINOX MN group, live/dead staining and Cell Titer Glo kit was employed to verify the organoid viabilities. As shown in Figure 4(c), the PC organoids showed spherical and hollow structures in the control and MN group, indicating the well proliferation and growth of the organoids [30]. In contrast, the organoids in the FOLFIRINOX MN group exhibited, respectively, irregular morphology, thinner wall structure, and more PI-stained cells, which demonstrated the unhealthy growth of the PC organoids. The quantified Cell Titer Glo results were consistent with the live/dead staining images (Figure 4(d)). The viability of the PC organoids in the FOLFIRINOX MN group started to decrease after 24 h coincubation with the FOLFIRINOX MNs and the cell viability continued to decrease for 72 h, suggesting that the chemotherapeutics could be release from the MNs sustainedly. These in vitro experimental results paved way for the in vivo evaluation of the therapeutic efficacy of the Christmas tree-shaped FOLFIRINOX MNs.

**Figure 5:**
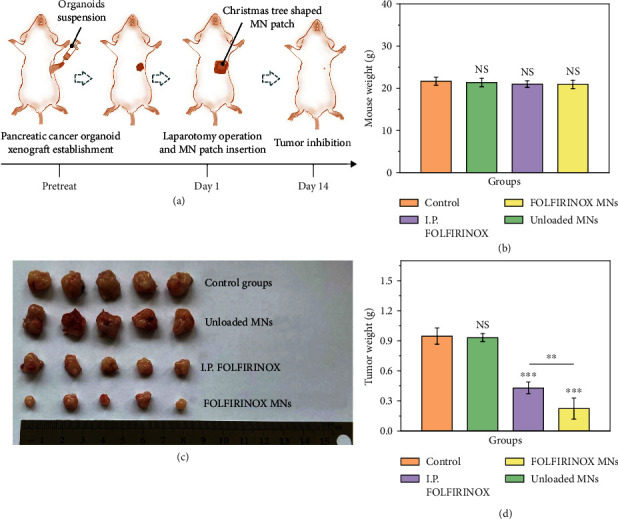
Anticancer efficacy of the FOLFIRINOX-loaded Christmas tree-shaped MN patch on patient-derived pancreatic tumor organoid xenografts. (a) Schematic flow gram of the in vivo experiments. (b) Mouse weight statistics from different groups. (c) Digital image of the pancreatic tumor organoid-derived tumor samples from different groups. (d) Statistical quantification of the tumor weights from different groups.

Encouraged by the results of in vitro experiments, we applied the FOLFIRINOX-loaded Christmas tree-shaped MN patch to in vivo experiments. To establish the pancreatic tumor organoid xenograft, the patient-derived PC organoid suspension was injected into the envelope of the pancreatic tail in mice. After the tumor grew to approximately 4 mm in diameter, the mice were randomly divided into 4 groups, namely, control group, unloaded MN group, intraperitoneally injecting (I.P.) FOLFIRINOX group, and FOLFIRINOX-loaded Christmas tree-shaped MN (FOLFIRINOX MN) group. For the control group, normal saline was injected intraperitoneally. For the unloaded MN group and FOLFIRINOX MN group, the MN patches were pressed onto the tumor surface after the laparotomy operation once a week (Figure 5(a)). The mice all survived after the insertion of MNs. For the I.P. FOLFIRINOX group, LV, L-OHP, 5-FU, and CPT-11 were mixed in 0.9% NaCl solution and injected through the tail vein immediately. The injection was performed once a week. After 21-day treatment, the mice all survived, and the weight of which showed no significant discrepancy (Figure 5(b)), indicating the nontoxicity of the MN patch. Additionally, the hematoxylin and eosin (H&E) staining of the main organs from mice treated with different regimens also suggested the biosafety of the MNs (Figure S4). According to the morphology and weight of the harvested tumor samples illustrated in Figures 5(c) and 5(d), it was demonstrated that the unloaded MN group (0.93 ± 0.04 g) showed no tumor inhibitory effect comparing to the control group (0.95 ± 0.08 g). Consistently, the intraperitoneal injection of FOLFIRINOX showed certain tumor killing effect (0.43 ± 0.06 g). Notably, the FOLFIRINOX MNs showed the significant elimination of tumors (0.22 ± 0.11 g).

**Figure 6:**
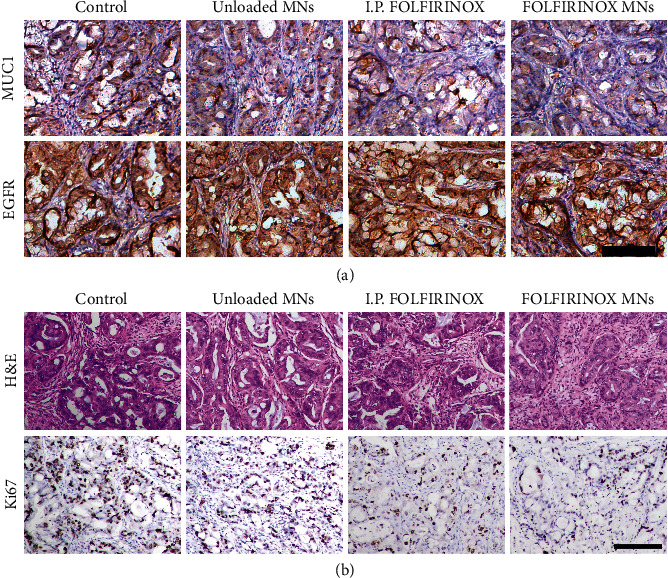
Histological analysis of the therapeutic efficacy of the FOLFIRINOX-loaded Christmas tree-shaped MN patch. (a) The MUC1 and EGFR immunohistology staining of the pancreatic tumor organoid xenograft samples from each group. (b) The representative H&E and Ki67 staining images of the organoid-derived tumor samples from different groups. Scale bars are 50 *μ*m in (a) and (b).

Through the immunohistology staining of MUC1 and EGFR, the successful establishment of PC organoid xenograft models can be proved (Figure 6(a)). The massive expression of MUC1 and EGFR demonstrated the purity of cancer cells in the organoid-derived xenograft model. Since desmoplasia is augmented in pancreatic tumors, the delivery of chemotherapeutics targeting PC is severely held up by the stromal tissue within the tumor. Attributing to the previous transdermal ability of MNs, the Christmas tree-shaped MNs could pierce the tumor tissue and deliver drugs into the tumor directly. Moreover, the traditional administrations of chemotherapeutics, such as intravenous injection and intraperitoneal injection, always lead to the off-target events and unwanted systematic side effects. However, the in situ application of drug-loaded MNs could significantly avoid these problems, contributing to less adverse effects and enhanced chemotherapeutic concentrations. The desired antitumor effect can be demonstrated by the H&E staining and immunohistology staining of Ki67 (Figure 6(b)). Compared with the control group and the unloaded MN group, the tumor tissue in the I.P. FOLFIRINOX group has less tumor cells and more collagen. Especially, only few tumor cells can be observed in the tumor tissue in the FOLFIRINOX MN group, indicating the excellent tumor eradication ability of FOLFIRINOX MNs. In addition, the proportion of Ki67-positive cells in the FOLFIRINOX MN group (9.0 ± 2.4%) was significantly less than that in the other three groups, suggesting the decreased proliferation ability of tumor cells (Figure 6(b) and Figure S5).

## 3. Discussion

Therefore, inspired by the hierarchical microarchitecture of the stings of wasps, we have presented a Christmas tree-shaped MN patch. This MN patch, compared with traditional single-layer MN patch, has shown enhanced adhesive property. By loading L-OHP and LV encapsulated in the top MNs and loading CPT-11 and 5-FU encapsulated in the bottom MNs simultaneously, the Christmas tree-shaped MNs could realize the FOLFIRINOX therapy in a single regimen. Through the in vitro experiments, we elucidated that the drugs loaded in different layers constructed with different hydrogels could release in batches, simulating the clinically employed FOLFIRINOX treatment. Noteworthy, the local application of the FLOFIRINOX-loaded MN patch avoided the systematical adverse effects which can be caused by the intravenous injection. In addition to the excellent biocompatibility of PEGDA hydrogel, our FLOFIRINOX-loaded Christmas tree-shaped MN patch could achieve an improved therapeutic method. Thus, it is convinced that MNs with Christmas tree-shaped structures could be promising candidates in biomedical application.

## 4. Materials and Methods

### 4.1. Materials and Organoids

Poly (ethylene glycol) diacrylate (PEGDA), gelatin methacryloyl (GelMA), 2-hydroxy-2-methylpropiophenone (HMPP), oxaliplatin (L-OHP), and CellTiter-Glo Luminescent Cell Viability Assay Kit was purchased from Sigma-Aldrich. Fluorouracil (5-FU), irinotecan (CPT-11), and leucovorin (LV) were purchased from Aladdin. The porcine skin was purchased from the market. Dulbecco's modified Eagle's medium (DMEM), penicillin/streptomycin (P/S), fetal bovine serum (FBS), Trypsin-EDTA solution, and phosphate buffer saline (PBS) were purchased from Gibco. The Live & Dead Viability/Cytotoxicity Assay Kit was purchased from Keygen Biotech Company. Pancreatic cancer surgical specimens were collected at the Department of General Surgery, Affiliated Drum Tower Hospital of Nanjing University Medical School. Male NSG mice aged 6-7 weeks were obtained from the Model Animals Research Center of Nanjing University. All animal treatments were operated in strict accordance with the guidelines approved by the Animal Ethics Committee of Nanjing Drum Tower Hospital.

### 4.2. Fabrication and Characterization of the Christmas Tree-Shaped MN Patch

For the top MNs, 10% (*v*/*v*) GelMA and 0.5% (*v*/*v*) HMPP were mixed together with L-OHP and LV solution to serve as the pregel solution (solution A). For the bottom MNs, 20% (*v*/*v*) PEGDA and 0.5% (*v*/*v*) HMPP were mixed with CPT-11 and 5-FU solution to serve as the pregel solution (solution B). For the MN patch, 20% (*v*/*v*) PEGDA and 0.5% (*v*/*v*) HMPP were mixed together to serve as the pregel solution (solution C). The abovementioned solutions were used right after they were prepared. A negative mold (Wisecare Corp, China), the tip depth of which was 500 *μ*m and the side length of the base patch of which was 1 cm, was applied. Solution B was first added into the mold. After 5 times of the vacuum process, the air in the tips was removed and the pregel solution filled into the tips. Then, the residue solution B was removed, and ultraviolet (UV) light was applied 30 s to polymerize PEGDA, followed by the readding and 30 s UV polymerization of solution C. Thus, a one-layer PEGDA MN patch can be obtained by squeezing the MN patch out of the negative mold. To fabricate the second layer, solution A was added into the mold and vacuumed for 5 times. After removing the residue solution A, the one-layer PEGDA MN patch was gently placed into the mold. By polymerizing solution A with 60 s UV, the double-layer MN patch can be obtained. The morphologic features of the Christmas tree-shaped MN was characterized with a stereomicroscope (JSZ6S, Jiangnan novel optics) equipped with a CCD camera (Oplenic digital camera).

### 4.3. Comparison of the Detachment Force between Traditional MN Patch and Christmas Tree-Shaped MN Patch

The porcine skin was washed and cut into portions to investigate the adhesion capacity of MN patch. A traditional MN patch with one layer of MNs was fabricated. The traditional MN patch and Christmas tree-shaped MN patch were placed onto two porcine skins and suppressed with a finger for 2 min with the same pressure. A custom-built tensile testing equipment was applied to measure the peeling-off strength between the MN patch and porcine skin. The patches were peeled off from one side slowly, the movement speed of which was 0.3 mm/s. The peeling-off tests were replicated for 5 times, and the peeling strength was recorded for further analysis.

### 4.4. Characterization of the Spatiotemporal Drug Delivery Property of Multi-Drug-Loaded Christmas Tree-Shaped MN Patch

To investigate the multi-drug-loading capacity of the Christmas tree-shaped MNs, 0.1 mg/mL red fluorescent dye was mixed with the PEGDA pregel solution to fabricate the top MNs, and 0.1 mg/mL blued fluorescent dye was mixed with PEGDA pregel solution to fabricate the bottom MNs. Through the bright-field, red, and blue fluorescent microscope (Carl Zeiss, Germany) observation, the different and well-defined red and blue fluorescence verified the multidrug spatial delivery property of the Christmas tree-shaped MNs. To investigate the temporal drug release capacity of the Christmas tree-shaped MNs, 0.1 mg/mL LV was mixed with the PEGDA pregel solution to fabricate the top MNs, and 1 mg/mL 5-FU was mixed with PEGDA pregel solution to fabricate the bottom MNs. Then, the fabricated MN patches loaded with merely LV in the top part or 5-FU in the bottom part were immersed into 37°C PBS. At certain time intervals, 200 *μ*L solution was extracted from the centrifuge tube and the absorbance of the detected with a plate reader (SpectraMax M3, USA). The release amount of LV was measured by detecting the absorbance at 281 nm, and the release amount of 5-FU was measured by detecting the absorbance at 265 nm. The measured solution was returned into the testing centrifuge tube. This experiment replicated three times.

### 4.5. Establishment and Culture of Patient-Derived Pancreatic Tumor Organoid

Organoids were established from fresh tumor tissue resected from PC patients. This experiment was approved by the Ethics Committee of Nanjing Drum Tower Hospital (No. 2020-072-01). Tumor tissues were minced into pieces and then digested with collagenase and DNAse mixture for 30 min. After washing with DMEM twice, the cells were centrifuged. Then, the cells were resuspended with Matrigel and seeded into a 6-well plate. The organoid culture medium was added to the wells after the Matrigel was fully solidified in 37°C. The organoid culture medium was composed of 50% Wnt3A conditioned media, 10% R-spondin1 conditioned media, 36% DMEM/F12 media, 0.01 *μ*mol/L Gastrin I, 500 nmol/L A83-01, 50 ng/mL EGF, 100 ng/mL Noggin, 100 ng/mLFGF-10, B27 supplement, and Glutamax.

### 4.6. In Vitro Cytotoxicity

Pancreatic cancer organoids were digested into single cells, and 4000 cells in 50 *μ*L Matrigel were seeded into each well of 24-well plates. The organoids were growing in organoid culture medium. 7 days later, when the cells formed the balloon morphology and reached a fast-growing state, MNs with or without FOLFIRINOX were used to treat the organoids by gently inserting the Christmas tree-shaped MN patch onto the surface of organoid-containing Matrigel. After another 24 h, the organoids were stained a live/dead staining kit. Also, the viability of organoids was examined using a CellTiter-Glo kit after 24, 48, and 72 h.

### 4.7. In Vivo Anticancer Efficacy

For organoid-derived xenograft model, 6- to 8-week-old male NSG mice were used in our experiment. Organoids were digested into single cells, and 106 cells were implanted orthotopically into the pancreas of each mouse. Two months later, the tumor volume reached about 4 mm and the mice were divided into four groups: (1) control group; (2) unloaded MN group; (3) I.P. FOLFIRINOX group; and (4) FOLFIRINOX MN group. For the I.P. FOLFIRINOX group, 24 mg/kg LV, 2 mg/kg L-OHP, 20 mg/kg 5-FU, and 20 mg/kg CPT-11 were mixed in 0.9% NaCl solution and injected through the tail vein immediately. The injection was performed once a week. For the MN groups, the mice were anesthetized, and the abdominal cavity was opened to press the MN patch onto the tumor. The surgery was also performed once a week. The loading amount of drugs in the FOLFIRINOX MN group was the same with that in the I.P. FOLFIRINOX group. After another 3 weeks, the mice were sacrificed, and the tumor weight was measured. The tumor tissues were also collected, and the immunohistochemistry analyses were performed to examine the proliferation index.

## Data Availability

The data that support the findings of this study are available in the supplementary material of this article.

## Supplementary Materials

Supplementary Materials Figure S1: the negative MN model and the fabricated MN patch. Figure S2: bright-field image, fluorescent images, and merge images of the Christmas tree-shaped MN loaded with two fluorescent dyes. Figure S3: biocompatibility of the Christmas tree-shaped MN patch. Figure S4: H&E staining of the main organs from different groups. Figure S5: the application process of the MN patch. Figure S6: quantification of the Ki67-positive cells.
