# Supplementary material for: Christmas Tree-Shaped Microneedles as FOLFIRINOX Spatiotemporal Delivery System for Pancreatic Cancer Treatment
Source: Research (Wash D C). 2022 Oct 16;2022:9809417. doi: 10.34133/2022/9809417 (PMC9620638; doi:10.34133/2022/9809417)
Supplement: Supplementary Materials — Figure S1: the negative MN model and the fabricated MN patch. Figure S2: bright-field image, fluorescent images, and merge images of the Christmas tree-shaped MN loaded with two fluorescent dyes. Figure S3: biocompatibility of the Christmas tree-shaped MN patch. Figure S4: H&E staining of the main organs from different groups. Figure S5: the application process of the MN patch. Figure S6: quantification of the Ki67-positive cells. [file 9809417.f1.docx]

Supplementary Materials

Fig. S1. The negative MN model and the fabricated MN patch.

Fig. S2. Bright field image, fluorescent images and merge images of the Christmas tree-shaped MN loaded with two fluorescent dyes.

Fig. S3. Biocompatibility of the Christmas tree-shaped MN patch.

Fig. S4. H&E staining of the main organs from different groups.

Fig. S5. The application process of the MN patch.

Fig. S6. Quantification of the Ki67 positive cells.


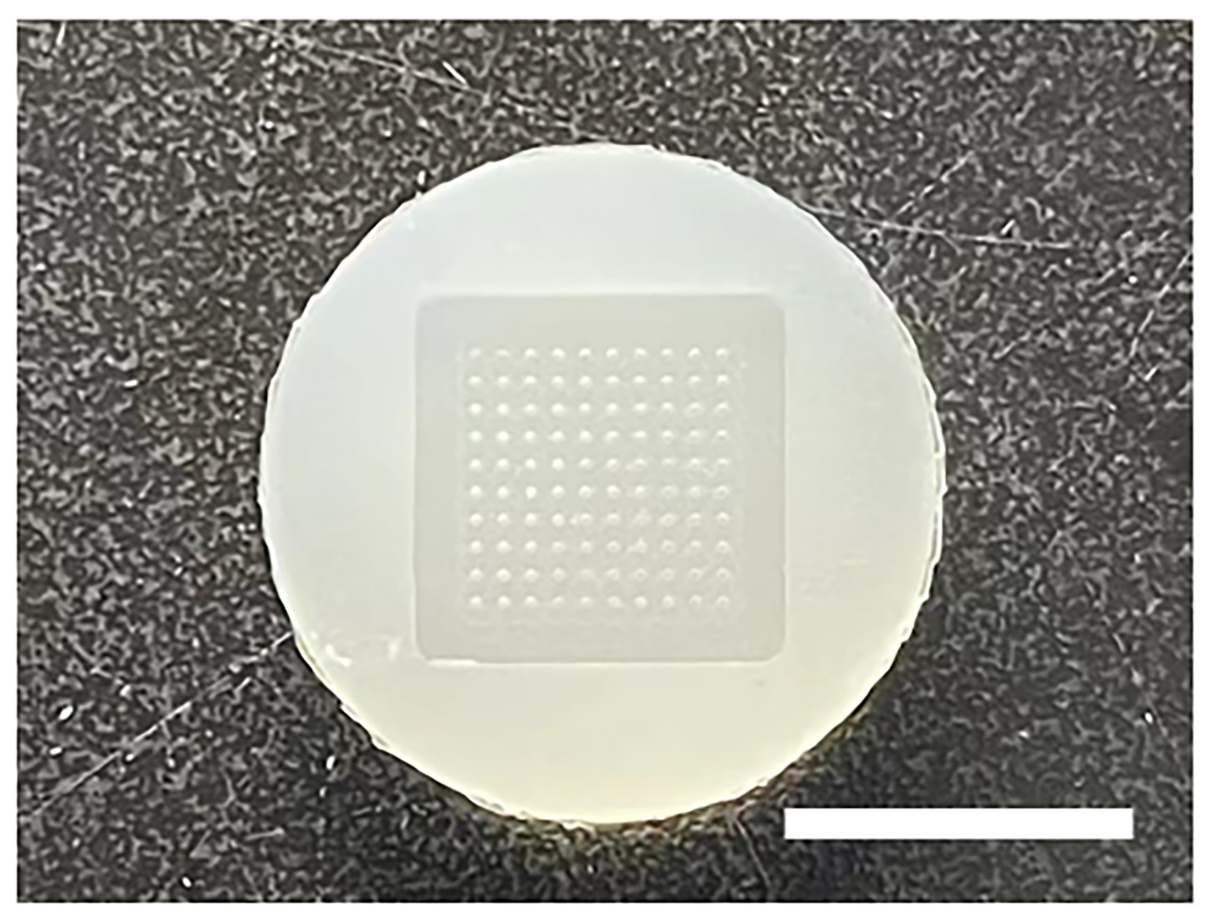


Figure S1. The negative MN model and the fabricated MN patch. Scale bar is 1 cm.


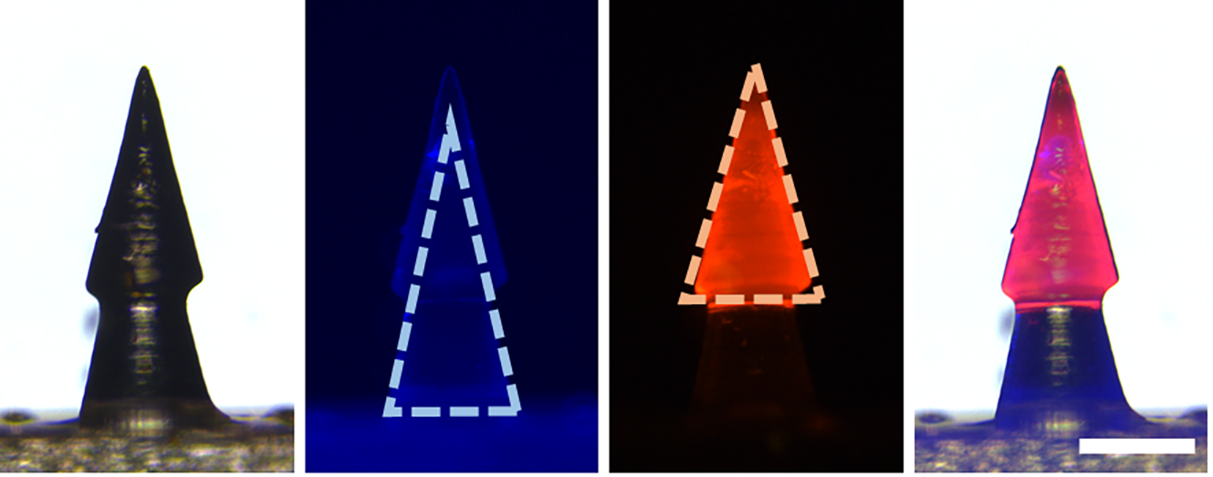


Figure S2. Bright field image, fluorescent images and merge images of the Christmas tree-shaped MN loaded with two fluorescent dyes. Scale bar is 300 μm.


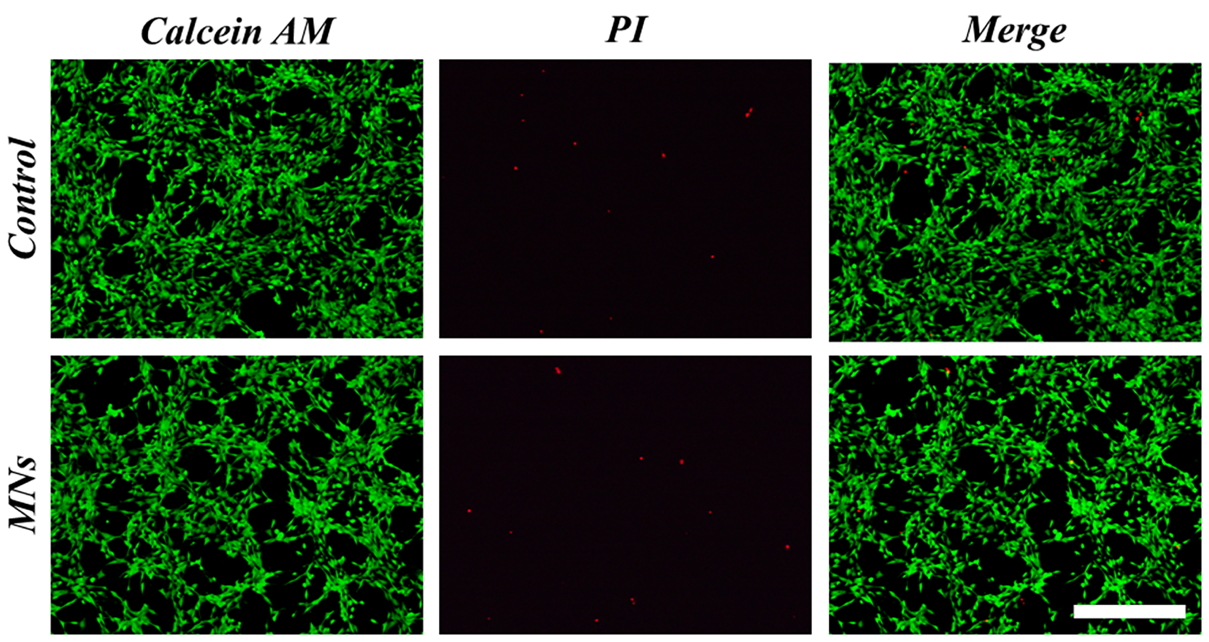


Figure S3. Biocompatibility of the Christmas tree-shaped MN patch.


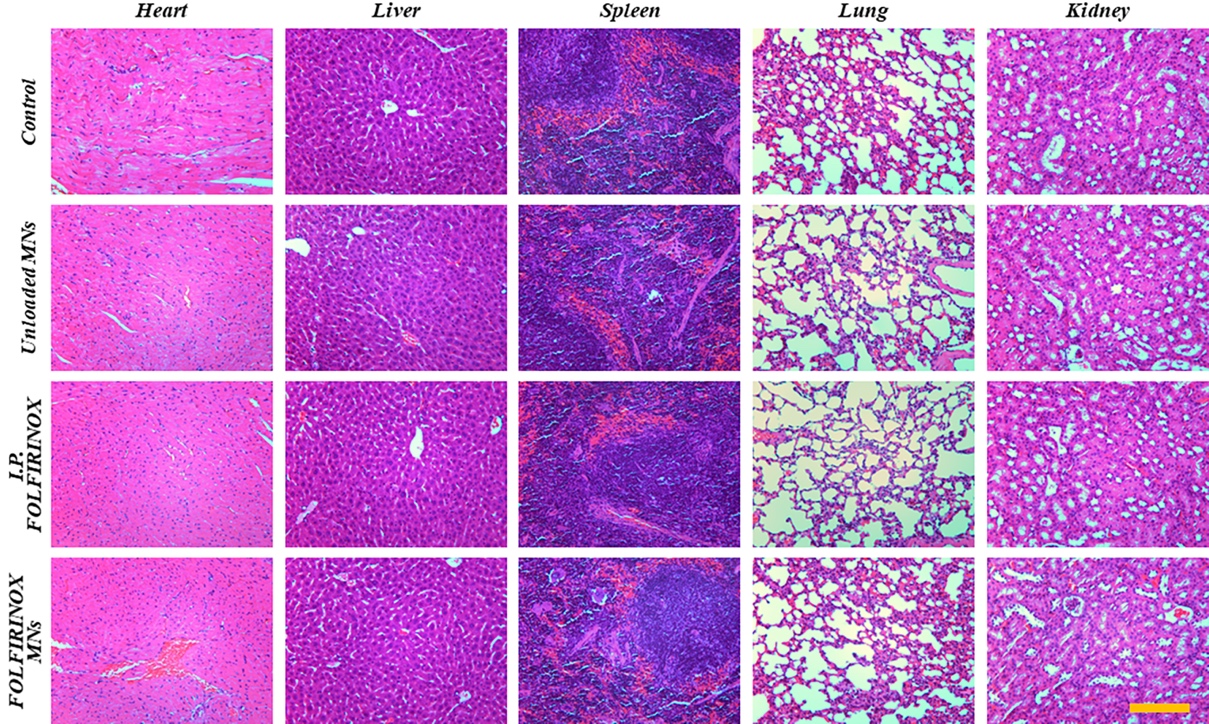


Figure S4. H&E staining of the main organs from different groups.


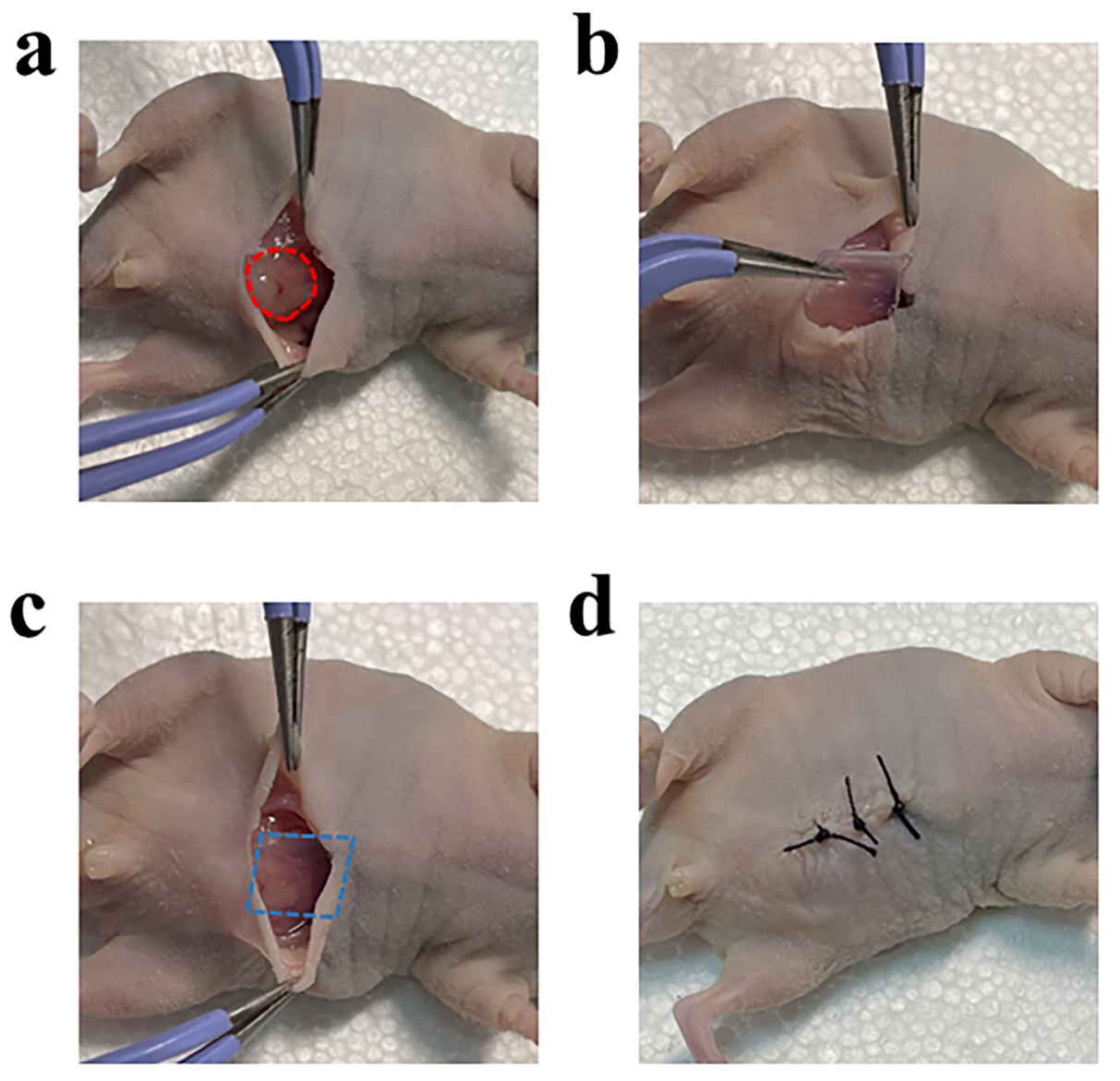


Figure S5. The application process of the MN patch. (a) The tumor was located after laparotomy. (b,c) The MN patch was inserted. (d) Abdomen was closed.


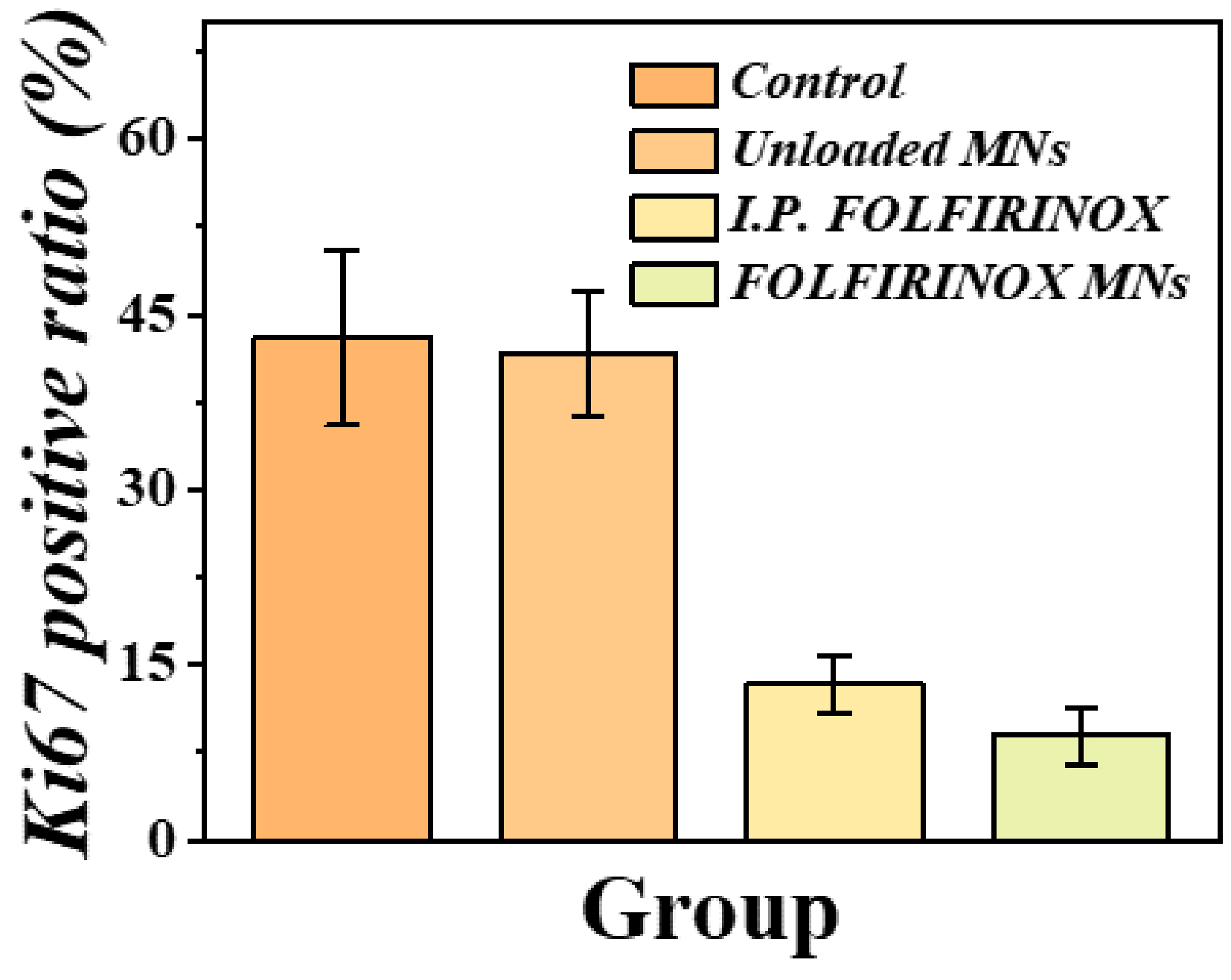


Figure S6. Quantification of the Ki67 positive cells.
